# BEAT: Beacon Inter-Reception Time Ensured Adaptive Transmission for Vehicle-to-Vehicle Safety Communication

**DOI:** 10.3390/s19143061

**Published:** 2019-07-11

**Authors:** Sunghwa Son, Kyung-Joon Park

**Affiliations:** Department of Information and Communication Engineering, Daegu Gyeongbuk Institute of Science and Technology (DGIST), Dalseong-gun, Daegu 42988, Korea

**Keywords:** vehicular ad hoc networks, vehicle-to-vehicle communications, safety beacon, congestion control, beacon inter-reception time

## Abstract

To improve vehicle safety, vehicular ad hoc networks (VANETs) periodically broadcast safety messages known as beacons. Consequently, it becomes safety critical to guarantee the timely reception of periodic beacons under the time-varying environments of VANET. However, existing approaches typically measure the packet delivery ratio, which is a time-average metric that does not consider the temporal behavior associated with beacon reception. In this paper, to properly reflect the temporal aspect of beacon reception, we propose a congestion control algorithm, Beacon inter-reception time Ensured Adaptive Transmission (BEAT). The proposed algorithm tightly regulates the beacon inter-reception time compared to conventional techniques, which can significantly improve vehicle safety. Our simulation results demonstrate the effectiveness of the proposed scheme.

## 1. Introduction

The Federal Communications Commission (FCC) has allocated licensed spectrum for dedicated short-range communication (DSRC) in the 5 GHz frequency band for intelligent transportation systems. Vehicles participating in vehicle-to-vehicle (V2V) communication must exchange the safety messages on the IEEE 802.11p control channel (CCH) for safety on the road. In Europe, the European Telecommunications Standards Institute (ETSI) has standardized the decentralized congestion control (DCC) algorithm to control periodic safety beacons based on the channel condition [1].

In VANET, two types of safety messages are exchanged for road safety. One of the safety messages is the event driven message, which is called emergency safety messages (ESMs). A vehicle generates the ESM when a potential danger is detected or when an emergency situation happens. In that situation, ESM should be disseminated with strict requirements of short delay and high reception ratio to other vehicles to notify an emergency situation. The congestion control may be preceded to meet the ESM requirements, but ESM is not the target of the congestion control. The other type of the safety messages in VANET is the periodic safety beacons, which are called basic safety messages (BSMs) in the US and cooperative awareness messages (CAMs) in Europe. These safety beacons are widely used in vehicular safety applications because they contain information such as position, speed, heading, acceleration, and brake status. While these periodic safety beacons contribute to vehicular safety on the road, there is the disadvantage that their use may lead to vehicular wireless channel congestion without proper congestion control.

Including the ETSI DCC algorithm, there is a substantial body of research on performance evaluation of decentralized congestion control for V2V safety communications. To mitigate congestion, most existing studies adapt the transmission parameters based on a time-average metrics such as the beacon reception rate (BRR) and the channel busy percentage (CBP) without properly considering the temporal aspect of individual beacon reception [2,3,4]. Meanwhile, there also exist research that considers the temporal behavior of beacon reception such as inter-reception time (IRT) related to awareness [5,6,7,8,9,10,11,12]. However, in these existing work, IRT is considered as a performance evaluation metric, and is not directly used to control the transmission parameters in real time.

In [2], the authors compared the application reliability of the message rate and the data rate congestion control algorithms, represented by LIMERIC and PDR-DCC, respectively. The application reliability is defined as the probability of the number of receiving beacons over a given period for a specific vehicular safety application. An integration of congestion and awareness control algorithm called INTERN is proposed in [3]. INTERN adapts the frequency margin based on channel busy percentage (CBP) of one and two hops and configures the transmission frequency to satisfy the requirements of the application and keep the channel load below the target CBP at the same time. In [4], the authors studied the performance of CAM depending on various parameters. A new statistical channel model is presented and the probability for a successful message reception with respect to the transmission distance is analyzed. The work in [5] proposes random transmission power selection to ensure a beacon reception for neighbors. By randomly selecting the transmission power, it can reduce the recurring collisions and improve the awareness quality at close range. The approach also reduces the channel congestion by transmitting less at longer distances. The authors showed that a reduction in the channel busy time ratio and in the probability for exceeding an update delay compared to constant full transmission power. In [6], the authors analyzed congestion control algorithms in terms of stability. They demonstrated the instability of the ETSI DCC and compared it with linear-adaptive congestion control through simulation. The channel load oscillations in the DCC are caused by the synchronized CBP measurement. The asynchronous CBP measurements are implemented as a continuous function for message rate for the ACTIVE states of DCC. For the performance evaluation, packet error rate (PER) and the 95th percentile inter packet gap (IPG) are used as metrics. To select important safety messages for verification in VANETs, the Relative-Time and Zone (RTZ) is proposed in [7]. The authors considered location and direction of the sender and relative-time between vehicles to provide higher awareness of nearby vehicles. The packet loss and the average inter-message delay are used as metrics to measure the awareness. The proposed scheme provides higher awareness of nearby vehicles with lower inter-message delay and reduced packet loss compared with other existing schemes. The authors of [8] evaluated the temporal beacon reception patterns in vehicular networks with packet inter-reception (PIR). They reported that the PIR time distribution is heavily tailed and the blackout probability is measured from the real-world experiments. However, they only used three vehicles in a highway scenario, thus did not consider the channel congestion. The authors of [9] presented a review of congestion control schemes for V2V communication. For the comparison of different approaches, they classified the congestion control algorithms in terms of type of traffic, control mechanism, and performance metrics. Some studies consider the IRT metric [10,11,12] but they are the averaged IRT value and used as performance metric not as control metric.

In this paper, we use the beacon inter-reception time as a control metric and propose an effective congestion control algorithm, namely Beacon inter-reception time Ensured Adaptive Transmission (BEAT), which can deliver safety beacons in a timely manner under dynamic environments.

The rest of this paper is organized as follows. Conventional congestion control algorithms are introduced in Section 2. The proposed algorithm based on the beacon inter-reception time is described in Section 3. In Section 4, simulation results are presented and we conclude this paper in Section 5.

## 2. Conventional Congestion Control Algorithms

First, we introduce two representative congestion control algorithms, DCC and LInear MEssage Rate Integrated Control (LIMERIC) [13]. They have the same property in the way they control the channel load based on measured CBP to avoid congestion. The difference resides in the congestion control function. The DCC is a state-based algorithm and works in a reactive manner, while LIMERIC works adaptively.

**Figure 1:**
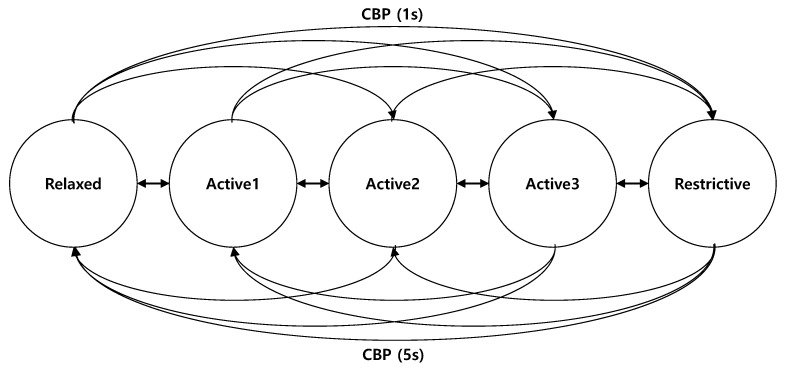
DCC state machine.

Figure 1 describes the state machine of the DCC algorithm. The DCC state machine has three states: relaxed, active, and restrictive. Notice that the active state can have several sub-states and the DCC maintains the number of active sub-states and the current state parameters [1]. We consider five states with three sub-active states in this paper. Each state has four control parameters: the transmission power, clear channel assessment (CCA) threshold, message frequency, and physical layer data rate.

Each of the four parameters are related to the DCC mechanisms, namely the transmit power control (TPC), the DCC sensitivity control (DSC), the transmit rate control (TRC), and the transmit data rate control (TDC). For congestion control of the periodic beacon, the DCC algorithm manages the aforementioned mechanisms by setting the values of four control parameters differently, as predefined for each state. The DCC algorithm employs the CBP as a condition for a state change by measuring the channel load and the current state depends on the current level of the CBP. As shown in Figure 1, the DCC state changes from relaxed to active or restrictive and from active to restrictive if the given condition of the CBP lasts for 1 s. Similarly, the DCC state changes from restrictive to active or relaxed and from active to relaxed when the CBP condition lasts for 5 s. The range of the channel load and the configuration of message frequency corresponding to each state we used in simulation is listed in Table 1.

In contrast to the DCC algorithm, LIMERIC operates in a linear and adaptive manner. Similar to the DCC algorithm, LIMERIC measures the channel load by using the CBP, but it only controls the message rate, which is periodically updated by the following equation:

$$\begin{array}{cc} r_{j}\left(t\right) & =\left(1-\alpha \right)r_{j}\left(t-1\right)+\beta \left(r_{g}-r_{C}\left(t-1\right)\right),\\ r_{C}\left(t\right) & =\sum_{j=1}^{K}r_{j}\left(t\right), \end{array}$$

where $r_{j}\left(t\right)$ is the message rate for the *j*th vehicle, *K* is the number of vehicles in the carrier sense range, $r_{C}$ is the rate for the sum of *K* vehicles, $r_{g}$ denotes the target total rate, and 0<α<1 and β>0 are gain factors [13].

However, to update the message rate, there is a premise condition of calculating $r_{C}$, which means that every single vehicle estimates *K* and shares its own rate with neighbors in its communication range. In practice, because of the dynamic varying environment of VANET, it is hard to estimate the number of neighboring vehicles in the carrier sense range. For the implementation of LIMERIC, it uses CBP to estimate $r_{C}$. At every 200 ms, the message rate is adapted between 1 and 10 Hz based on its previous value and the difference between the target and measured CBP.

## 3. Beacon Inter-Reception Time Ensured Adaptive Transmission (BEAT)

In this section, we briefly explain with a situational illustration the reason that the time-average metric is inadequate to cover the time-varying environment of V2V communication and the proposed scheme is described.

The conventional metrics such as CBP and BRR are insufficient to capture the temporal behavior of beacon reception. In fact, the CBP only tells the portion of the channel busy time occupied by other transmission or noise. Although the BRR measures the average rate of beacon reception, it is still insufficient to represent the temporal behavior of beacon reception. For example, let us consider the situation when the vehicle receives 50 beacons out of 100, which corresponds to BRR =0.5. However, there could be two extreme cases with the same BRR as follows: In the first case, the vehicle receives every other beacon, in which the beacon inter-reception time is equal to twice the beacon broadcasting period. In the second case, the vehicle receives the first 50 beacons and misses the rest, in which the inter-reception time becomes 50 times larger than the beacon broadcasting period. In summary, the beacon inter-reception time should be properly incorporated into congestion control for V2V safety communication.

To avoid an emergency situation such as crash, a vehicle participating in vehicle-to-vehicle communication has to receive a certain number of periodic safety beacons in real time. Both the conventional DCC and LIMERIC consider a time-average metric of CBP for a control metric. However, in V2V safety communication, it is more appropriate to take into account a metric that properly reflects the temporal behavior of each beacon reception. In addition, as discussed in [7], a vehicle cannot verify all the safety messages, thus an appropriate solution is needed to overcome the safety beacon overflow in VANET. Meanwhile, the inter-reception time can be measured at every reception and it contains the information of the reception behavior in real time and a time delay from the previous reception. To this end, we propose an effective congestion control algorithm called Beacon inter-reception time Ensured Adaptive Transmission (BEAT), which considers the *beacon inter-reception time (BIRT)* as a control metric.

Reflecting the temporal aspect of beacon reception is the main advantage of using BIRT as a control metric. To handle the congestion in V2V communication, knowing the capacity of wireless channel is essential and adjusting the transmission parameters to make the traffic to under the network capacity. Through the beacon reception behavior, a vehicle can indirectly guess whether the channel is congested. Moreover, because BIRT is measured whenever a beacon is received, it can rapidly react to the congestion. The proposed BEAT controls the message frequency based on BIRT to guarantee the timely beacon reception. To measure BIRT, every vehicle manages (vehicle ID, time) pairs whenever receiving a beacon. Then, BIRT is calculated by the time difference between two successive beacon receptions with respect to the unique vehicle ID. Algorithm 1 describes how BEAT controls the message frequency of Vehicle *i*. Based on the measured BIRT, each vehicle can estimate the channel congestion level and adjust the message frequency *f*, where f=1,2,3,⋯,10 Hz. The details of the proposed BEAT algorithm is as follows.

**Algorithm 1:** Pseudocode of BEAT

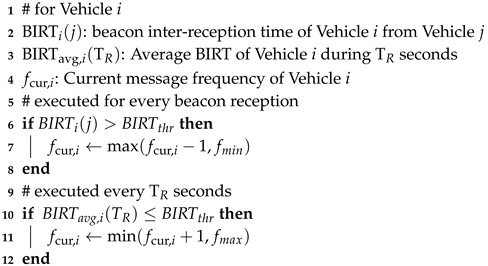



The condition on Line 6 of Algorithm 1 is responsible for decreasing the message frequency in case of congestion. First, we need to consider the tolerance time defined as the time duration during which at least one beacon should be received. Although varied in applications [14], we choose a typical value of 1 s as the tolerance time. Thus, to meet the tolerance time constraint, we set a BIRT threshold (${\mathrm{BIRT}}_{thr}$) to 1 s.

Since the BIRT threshold is set to a fixed value, the maximum allowable number of consecutive missing beacons is different with the message frequency. In the case of *k* consecutive missing beacons, BIRT becomes (k+1)/f. Considering the BIRT threshold, the number of maximum allowable consecutive missing beacons, $k_{m}$, with respect to the message frequency, $f_{i}$, is calculated as $k_{m}={\mathrm{argmax}}_{k}\left(\left(k+1\right)/f_{i}\leq {\mathrm{BIRT}}_{thr}\right)$.

For instance, the vehicle with the message frequency of 10 Hz has $k_{m}=9$. Hence, it can keep the current message frequency with up to nine consecutive beacons missing. If the vehicle misses more than $k_{m}$ consecutive beacons, then the vehicle should decrease its message frequency because BIRT exceeds ${\mathrm{BIRT}}_{thr}$ on Line 6 of Algorithm 1. Here, the max function holds the current message frequency not to be under the minimum message frequency, $f_{min}$. The decreasing message frequency event can occur before the next beacon reception of Vehicle *j* because the condition is checked for every nearby neighboring vehicle, not only Vehicle *j*. Thus, in the congested situation, the message frequency is decreased rapidly and the channel congestion can be resolved in a short time.

The condition on Line 10 of Algorithm 1 is for increasing the message frequency to improve the awareness of neighboring vehicles. Vehicle *i* checks the condition every $T_{R}$, which is set to 5 s, the same as in DCC. During this period, if the average BIRT is below or equal to the threshold, the vehicle increases its message frequency. Compared to the condition on Line 6, the condition on Line 10 is checked periodically and slowly every $T_{R}$ s. Even though the channel congestion is caused by the increased message frequency, the sensitive decreasing message frequency process can attempt to recover it at the moment of satisfying the condition on Line 6.

In this manner, the proposed BEAT algorithm directly controls the beacon inter-reception time.

## 4. Performance of BEAT

To evaluate the performance of BEAT, we carried out a simulation study using OMNeT++ [15] and SUMO [16]. We considered a highway scenario to highlight the issues of real time operation. In this scenario, the highway had four lanes with different velocity limits of 25, 30, 35, and 40 m/s, respectively. There were a total of 200 vehicles, and reference vehicle and observed vehicles were allocated on the 40 m/s lane. The rest of the vehicles were randomly distributed at a density of 50 vehicles/lane/km on the other three lanes make a dynamically changing vehicular environment around the observed vehicles. A Nakagami-m fading model was used for the wireless channel. We used Nakagami fading parameter m = 1, which refers to severe fading conditions [17]. Each simulation was conducted for 50 s and we ran a total of 100 simulations with independent random seeds per each congestion control algorithm. In summary, because vehicles were randomly positioned with independent seed, the number of neighbor vehicles varied across each simulation run. Consequently, the reference vehicle and the observed vehicles experienced different congestion, i.e., different wireless channel condition, every single simulation.

Table 2 shows the parameter settings for the BEAT, LIMERIC, and DCC algorithms. The PHY and MAC layers followed the IEEE 802.11p standard. The beacon was assigned to priority level 2, which is the second highest priority, and the same level of access category to video in enhanced distributed channel access (EDCA). The values of α, β, and the target CBP were from [13]. DCC, LIMERIC, and BEAT used identical settings for transmission power, carrier sense threshold, and data rate. These common parameter settings are widely adopted to the congestion control algorithms in vehicular communications and we obeyed the adjustment range standard [9]. In simulation, the message frequency started with 10 Hz initially and adapted with respect to the function of each algorithm. The DCC algorithm also set the message frequency and state change conditions as illustrated in Table 1.

For performance comparison of the DCC and LIMERIC algorithms with the proposed BEAT algorithm, we measured BIRT values of vehicles 50, 100, 150, 200, 250, and 300 m away from the reference vehicle.

**Figure 2:**
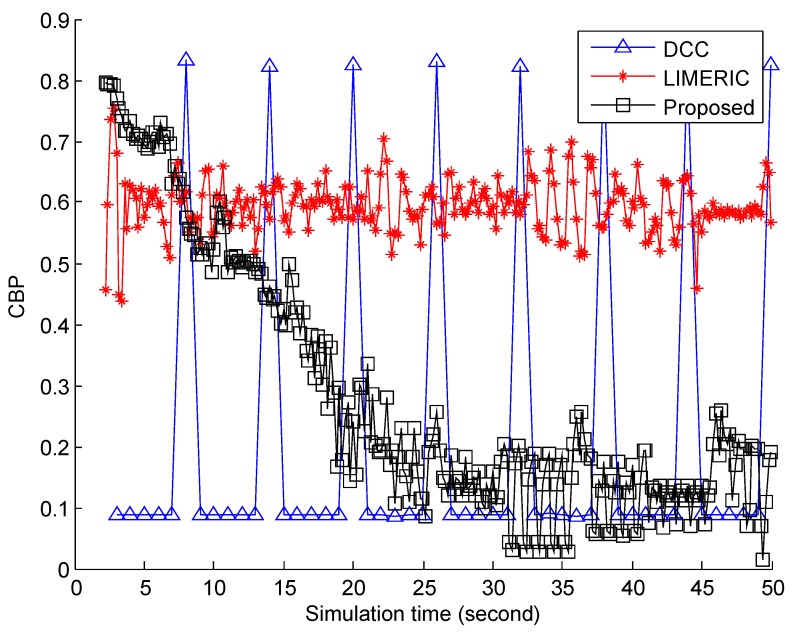
CBP comparison of the reference vehicle.

The comparison of the CBP among the DCC, LIMERIC, and BEAT algorithms is presented in Figure 2. The CBP of the DCC algorithm significantly oscillated because its state frequently changed between relaxed and restrictive. Regardless of the environment change in VANET, the CBP change pattern of the DCC was fixed and repeated: after staying 0.1 during 5 s, it peaked around 0.8 and then returned to 0.1. Meanwhile, the CBP of LIMERIC remained in a range of 0.5 and 0.7 because LIMERIC was basically a control algorithm to meet the target CBP of 0.65. The proposed scheme, BEAT, was distinct from DCC and LIMERIC. BEAT initially experienced high CBP because of a high message frequency. However, its CBP decreased to a small value by controlling the message frequency based on BIRT.

**Figure 3:**
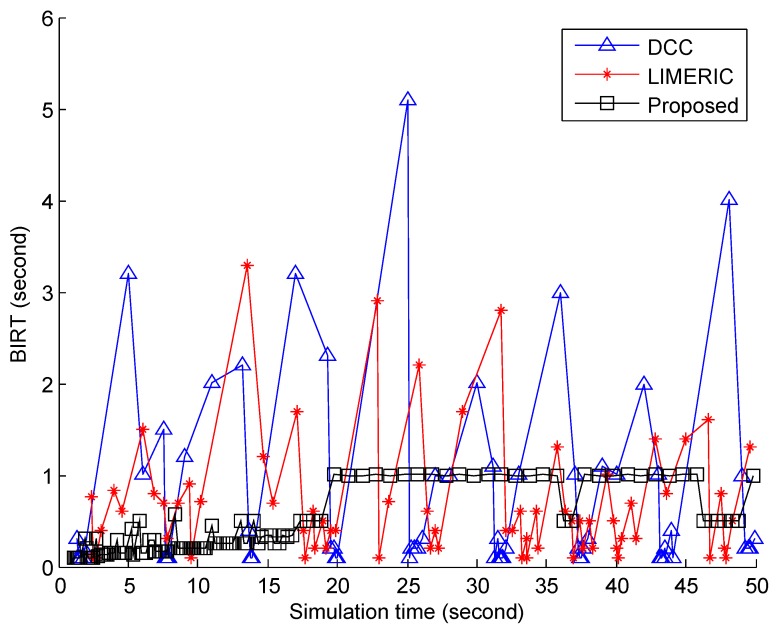
BIRT of a vehicle 50 m away from the reference vehicle.

Next, we checked the performance of the algorithms in terms of BIRT, which is critical to the safety performance. Figure 3 shows the BIRT value of a vehicle 50 m-away from the reference one. Despite the relatively short distance between the two vehicles, the DCC algorithm frequently failed to receive the beacon, and BIRT often increased up to around 5 s. Although smaller than DCC, BIRT of LIMERIC also often increased up to around 3 s. Considering the tolerance time of safety application in VANET, the long BIRT of DCC and LIMERIC could be a potential danger on the road. From the perspective of the vehicle, one of the neighboring vehicles might appear to vanish for up to 3 or 5 s. More seriously, not all the neighboring vehicles, but some of them may be found in this situation and suffer because the nearby vehicles experience similar channel conditions. This problem makes the vehicle make a wrong decision from lack of information and cause vehicle crash in the worst case. For the safety on the road, guaranteeing the timely reception of the safety beacon cannot be neglected. In comparison to the DCC algorithm and LIMERIC, the proposed BEAT algorithm tightly controlled the message frequency and was therefore able to keep BIRT under 1 s. The trends are similar for other distances.

**Figure 4:**
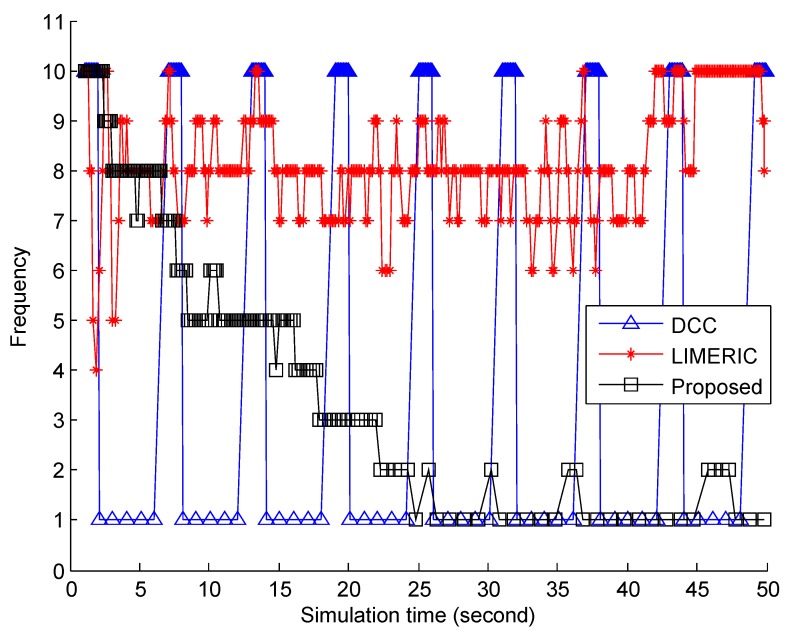
Comparison of beacon frequency of the three congestion control algorithms.

Figure 4 compares the beacon frequency of the three congestion control algorithms, DCC, LIMERIC, and BEAT. The DCC algorithm controlled the message frequency in accordance with the CBP, as shown in Figure 2. As a result, the message frequency of the DCC algorithm oscillated between 1 and 10 by repeating the same process again as in the CBP graph. LIMERIC also controlled the message frequency to meet the target channel load in the range between 4 and 10. Unlike DCC and LIMERIC, BEAT adapted its message frequency according to the measured BIRT rather than CBP. Consequently, similar to the results shown in Figure 2, the message frequency of BEAT decreased smoothly and remained around 1∼2 Hz. In Figure 4, the main difference of the proposed BEAT and the conventional schemes is the average message frequency. The high message frequency of the conventional algorithms is expected by Figure 2. Generally, the high message frequency is related to the high throughput and it means that the conventional algorithms may have greater throughput than BEAT. However, the objective of this paper is the congestion control, which can react properly in real time for vehicle-to-vehicle safety communication, not to improve the throughput.

**Figure 5:**
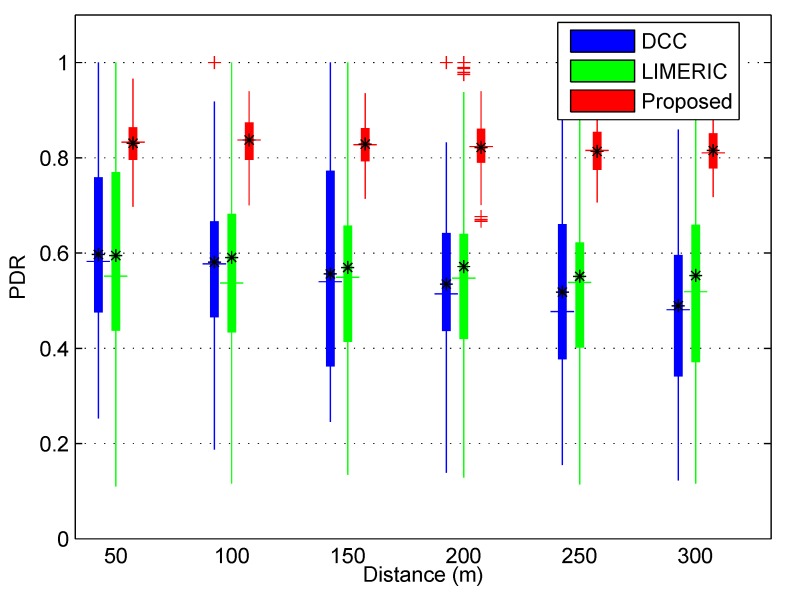
Packet delivery ratio (PDR) performance of the three congestion control algorithms.

We also measured the average performance metric, packet delivery ratio (PDR), as illustrated in Figure 5. The PDR results of each simulation is displayed as a type of box plot according to the distance from the reference vehicle. In the box, the horizontal line is the median, the asterisk is the average value of PDR, the plus signs are outliers, and the bottom and top edges of the box indicate the 25th and 75th percentiles, respectively. As shown in the figure, the PDR values of BEAT are closely gathered, while the DCC and LIMERIC are widely distributed. Moreover, the observed vehicles of BEAT received over 80% of safety beacons on average from the reference vehicle. The average PDR of BEAT was the highest compared to the conventional congestion control algorithms in the entire distance range. We observed that the DCC and LIMERIC have high message frequency and long BIRT values (Figure 3 and Figure 4). Additionally, the extreme deviation of the PDR value at the same distance means unfair safety beacon reception between the vehicles and it may cause a safety hazard in a real scenario. However, the distribution of the PDR value shows BEAT received safety beacons fairly from all vehicles in comparison to the conventional algorithms.

**Figure 6:**
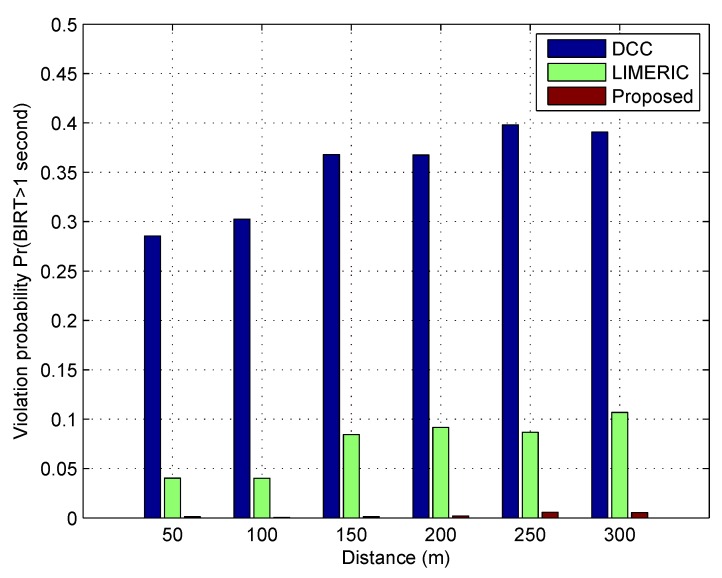
Violation probability of BIRT.

Figure 6 shows the violation probability of each algorithm according to the distance from the reference vehicle, where the violation condition is that BIRT is greater than 1 s. The violation probability is defined as follows.

$$\mathrm{Violation}\mathrm{probability}=\frac{\mathrm{The}\mathrm{number}\mathrm{of}\mathrm{received}\mathrm{beacons}\mathrm{with}\mathrm{BIRT}\mathrm{exceeds}\;1\;s}{\mathrm{Total}\mathrm{number}\mathrm{of}\mathrm{received}\mathrm{beacons}}.$$

In terms of the violation probability, the DCC algorithm shows the worst performance. Regardless of the distance, BEAT showed extremely low violation probability of less than 0.006. The reason the DCC had high violation probability value is the poor performance of BIRT. The violation probability was calculated only for the received safety beacons. For instance, the frequency behavior of the DCC for 10 s shown in Figure 4 has a restrictive state (1 Hz) at 1–5 and 7–10 s and relaxed state (10 Hz) at 6 s. If the worst BIRT situation occurred at relaxed state, the DCC operated vehicle would lose 10 and 3 safety beacons at relaxed and restrictive states, respectively. However, the violation probability could be calculated to 1/6.

## 5. Conclusions

In this paper, we discuss the importance of safety beacon inter-reception time as a performance metric. We focus on the real time property of the vehicle-to-vehicle communication, thus adopt BIRT, which can reflect the temporal aspect of beacon reception, rather than BRR and throughput. Then, we propose a congestion control scheme named BEAT. The message frequency of periodic safety beacon is adjusted based on BIRT to guarantee the timely beacon reception. Our simulation results demonstrate that BEAT is more effective for timely delivery of safety beacons than the conventional DCC and LIMERIC algorithms. We expect that BEAT will contribute to V2V safety communication. We are interested in the following directions for future work. The scalability of BEAT with respect to the vehicle density and different scenarios would be considered. In the proposed algorithm, in addition to BIRT, the distance between neighboring vehicles may contribute to improve the awareness level.

## Figures and Tables

**Table 1:** Configuration of CBP and frequency values to the state.

State	CBP Condition	Frequency
Relaxed	<0.3	10 Hz
Active 1	0.3–0.39	5 Hz
Active 2	0.4–0.49	2.5 Hz
Active 3	0.5–0.59	2 Hz
Restrictive	>0.6	1 Hz

**Table 2:** Parameter setting of simulation environment.

Parameter	Value
AIFSN (priority)	2 (2)
$CW_{min}$	15
Beacon length	378 byte
Transmission power	20 dBm
Carrier sense threshold	−92 dBm
Noise floor	−110 dBm
Data rate	6 Mbps
Wireless channel	Nakagami-m fading
CBP update period	200 ms
**LIMERIC Parameter Setting**	
α	0.1
β	1/150
Target CBP	65%
**DCC Parameter Setting**	
State change period	1 or 5 s

## References

[1] European Telecommunications Standards Institute (ETSI) (2011). ETSI-TS 102 687, Intelligent Transport Systems (ITS), Decentralized Congestion Control Mechanisms for Intelligent Transport Systems Operating in the 5 GHz Range, Access Layer Part, V1.1.1.

[2] Math C.B., Li H., de Groot S.H., Niemegeers I.G. (2017). V2X Application-Reliability Analysis of Data-Rate and Message-Rate Congestion Control Algorithms. IEEE Commun. Lett..

[3] Sepulcre M., Gozalvez J., Altintas O., Kremo H. (2016). Integration of Congestion and Awareness Control in Vehicular Networks. Ad Hoc Netw..

[4] Breu J., Brakemeier A., Menth M. (2014). A Quantitative Study of Cooperative Awareness Messages in Production VANETs. EURASIP J. Wirel. Commun. Netw..

[5] Kloiber B., Harri J., Strang T. Dice the TX Power-Improving Awareness Quality in VANETs by Random Transmit Power Selection. Proceedings of the 2012 IEEE Vehicular Networking Conference.

[6] Rostami A., Cheng B., Bansal G., Sjöberg K., Gruteser M., Kenney J.B. (2016). Stability Challenges and Enhancements for Vehicular Channel Congestion Control Approaches. IEEE Trans. Intell. Transp. Syst..

[7] Banani S., Gordon S., Thiemjarus S., Kittipiyakul S. (2018). Verifying Safety Messages Using Relative-Time and Zone Priority in Vehicular Ad Hoc Networks. Sensors.

[8] Renda M.E., Resta G., Santi P., Martelli F., Franchini A. (2016). IEEE 802.11p VANets: Experimental Evaluation of Packet Inter-Reception Time. Comput. Commun..

[9] Liu X., Jaekel A. (2019). Congestion Control in V2V Safety Communication: Problem, Analysis, Approaches. Electronics.

[10] Ruehrup S., Fuxjaeger P., Smely D. TCP-Like Congestion Control for Broadcast Channel Access in VANETs. Proceedings of the 2014 International Conference on Connected Vehicles and Expo (ICCVE).

[11] Joseph M., Liu X., Jaekel A. An Adaptive Power Level Control Algorithm for DSRC Congestion Control. Proceedings of the 8th ACM Symposium on Design and Analysis of Intelligent Vehicular Networks and Applications.

[12] Tielert T., Jiang D., Hartenstein H., Delgrossi L. Joint Power/Rate Congestion Control Optimizing Packet Reception in Vehicle Safety Communications. Proceedings of the Tenth ACM International Workshop on Vehicular Inter-Networking, Systems, and Applications.

[13] European Telecommunications Standards Institute (ETSI) (2014). ETSI-TR 101 612, Intelligent Transport Systems (ITS), Cross Layer DCC Management Entity for Operation in the ITS G5A and ITS G5B Medium, Report on Cross Layer DCC Algorithms and Performance Evaluation, V1.1.1.

[14] Bai F., Krishnan H. Reliability Analysis of DSRC Wireless Communications for Vehicle Safety Applications. Proceedings of the 2006 IEEE Inelligent Transportation Systems Conference.

[15] OMNet Web Site. http://omnetpp.org.

[16] Krajzewicz D., Erdmann J., Behrisch M., Bieker L. (2012). Recent Development and Applications of SUMO-Simulation of Urban Mobility. Int. J. Adv. Syst. Meas..

[17] Torrent-Moreno M., Mittag J., Santi P., Hartenstein H. (2009). Vehicle-to-Vehicle Communication: Fair Transmit Power Control for Safety-Critical Information. IEEE Trans. Veh. Technol..

